# Heparin and Arginine Based Plasmin Nanoformulation for Ischemic Stroke Therapy

**DOI:** 10.3390/ijms222111477

**Published:** 2021-10-25

**Authors:** Ramsha Aamir, Cameron Fyffe, Netanel Korin, Daniel A. Lawrence, Enming J. Su, Mathumai Kanapathipillai

**Affiliations:** 1 Department of Mechanical Engineering, University of Michigan-Dearborn, Dearborn, MI 48128, USA; raamir@umich.edu (R.A.); cfyffe@umich.edu (C.F.); 2 Department of Biomedical Engineering, Technion-Israel Institute of Technology, Haifa 32000, Israel; korin@bm.technion.ac.il; 3 Department of Internal Medicine, University of Michigan Medical School, Ann Arbor, MI 48109, USA; dlawrenc@med.umich.edu

**Keywords:** plasmin, nanoformulation, ischemic stroke, fibrinolysis, heparin

## Abstract

Ischemic stroke is the most common type of stroke and thrombolytic therapy is the only approved treatment. However, current thrombolytic therapy with tissue plasminogen activator (tPA) is often hampered by the increased risk of hemorrhage. Plasmin, a direct fibrinolytic, has a significantly superior hemostatic safety profile; however, if injected intravenously it becomes rapidly inactivated by anti-plasmin. Nanoformulations have been shown to increase drug stability and half-life and hence could be applied to increase the plasmin therapeutic efficacy. Here in this paper, we report a novel heparin and arginine-based plasmin nanoformulation that exhibits increased plasmin stability and efficacy. In vitro studies revealed significant plasmin stability in the presence of anti-plasmin and efficient fibrinolytic activity. In addition, these particles showed no significant toxicity or oxidative stress effects in human brain microvascular endothelial cells, and no significant blood brain barrier permeability. Further, in a mouse photothrombotic stroke model, plasmin nanoparticles exhibited significant efficacy in reducing stroke volume without overt intracerebral hemorrhage (ICH) compared to free plasmin treatment. The study shows the potential of a plasmin nanoformulation in ischemic stroke therapy.

## 1. Introduction

Stroke is a major cause of death worldwide, affecting more than fifteen million people every year. There are two types of stroke, ischemic and hemorrhagic. Nearly 90% of strokes are ischemic and the majority of these are thrombotic [1]. Outcomes for hemorrhagic strokes are generally worse than for ischemic strokes [2] and hemorrhagic conversion of an ischemic stroke markedly increases stroke severity [3,4,5,6,7]. Since most strokes are ischemic, effective thrombolytic therapy is needed. Thrombolysis aids in the dissolution of blood clots and the restoration of blood flow limiting subsequent neuronal damage. Currently, tissue-type plasminogen activator (tPA) is the only approved thrombolytic drug. Intravenous delivery of recombinant tPA within 4.5h after symptom onset provided better reperfusion, independence, survival and more favorable outcomes. However, both clinical evidence, and experimental models of ischemic stroke, demonstrate a significant increase in the incidence of hemorrhagic transformation with late thrombolytic tPA beyond the 4.5h treatment window [8,9,10,11,12,13,14]. Recent studies indicate that tPA in the brain parenchyma increases the blood brain barrier (BBB) permeability, via a pathway that is regulated by the platelet derived growth factor receptor alpha on perivascular astrocytes and exacerbates the stroke outcome [15,16]. Thus, an alternative thrombolytic approach that could reduce tPA-mediated effects on the BBB might significantly improve the therapeutic outcome of thrombolytic therapy.

Plasmin, a direct fibrinolytic, has shown a safer and more effective thrombolytic profile compared to recombinant tissue plasminogen activator (rtPA) for the treatment of acute ischemic stroke [17,18,19]. However, if injected intravenously, it is rapidly inactivated by anti-plasmin and other plasmin inhibitors, and hence is not effective when used as a systemic therapeutic agent. Additionally, its sensitivity to environmental conditions, including pH and temperature, makes it challenging to preserve its activity [19]. Recently studies have been performed to formulate plasmin delivery by liposomal and protein carriers [20,21]. The studies were limited to in vitro clot lysis or micro-plasmin-based formulations. Hence, further improvements and detailed studies are needed in the field of plasmin nanoformulations, to realize superior thrombolytic properties with minimal side effects, for them to be clinically relevant. In theory, a plasmin carrier that could retain its activity in vivo and deliver it at the occlusion site would have improved therapeutic benefit.

Nanoformulations increase drug solubility and stability and can be tuned to deliver the cargo to a desired site in a controlled release manner, all of which can increase the therapeutic efficacy of a drug and minimize the systemic side effects [22,23]. Several studies have been reported on engineered nanoformulations to enhance fibrinolysis [24]. Previously we have developed shear-activated microparticles for clot dissolution [25]. When coated with tPA, these aggregates effectively dissolved partially occluded clots at the diseased site and showed several-fold increases in efficacy compared to the free tPA treatment. All these applications of nano/micro formulations indicate their potential as effective drug depots for thrombolytic drug delivery. Hence, a nanoparticle that could shield the plasmin from the external environment, deliver plasmin to the stroke area, but not cross the BBB, could be an ideal candidate for plasmin delivery.

In this paper, we utilize a nanotechnology-based approach that could address the plasmin inactivation during stroke therapy. For this study, the nanoparticles were formed using biodegradable and biocompatible heparin/polyarginine based polymers, which have been widely used in biomedical applications [26,27] and been shown to be safe in vivo, as our base material for the nanoparticle formulation. Heparin is known to have anticoagulant properties [28] while arginine is known to facilitate plasmin stability and increase its activity [19]. In addition, it has been reported that arginine plays a role in inhibiting hemostasis activation [29]. Further, the anionic and cationic nature of the heparin and arginine helps in the formation of nanocomplexes and shields the plasmin from the external environment. This manuscript reports the production of the heparin-arginine based formulation, physicochemical chemical characterization, in vitro efficacy studies, and in vivo efficacy studies in a mouse photothrombotic model. The study shows the potential of nanoformulations for plasmin delivery and could open new paths for efficient plasmin-based stroke therapy. 

## 2. Results

**Figure 1:**
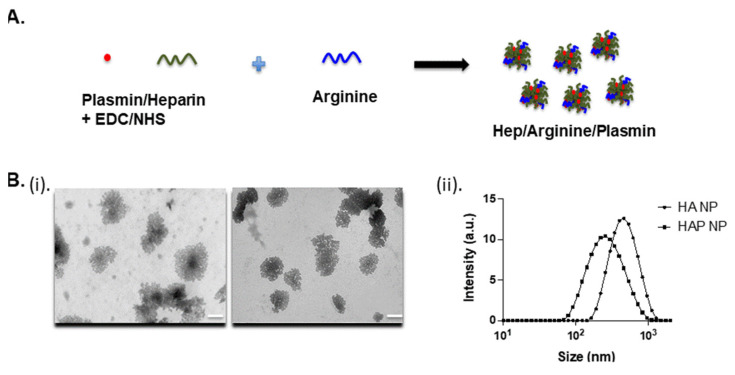
(**A**) Schematic of the heparin/arginine/plasmin (HAP) nanocomplex formation. Heparin was first reacted with plasmin and subsequently complexed with polyarginine. (**B**) (i) Transmission electron microscopy images of the nanoparticles showing the morphology and size range of the nanoparticles with and without plasmin. Scale bar 200 nm. (ii) Size and zeta potential characterization of the plasmin formulation. Dynamic light scattering and zeta measurements revealed sizes around 370–470 nm of the heparin-arginine complex with and without plasmin.

The heparin-arginine based plasmin nanoparticles were produced by reaction in the presence of EDC/NHS in HEPES buffer, as shown in Figure 1A. The plasmin nanocomplex was formed due to the charge interaction of heparin and arginine and the crosslinking of amine groups. The transmission electron microscopy (TEM) and dynamic light scattering (DLS) measurements revealed particles in the range of 370–470 nm with or without plasmin. The heparin-arginine nanoparticles (HA NP) exhibited larger size compared to the heparin-arginine-plasmin nanoparticles (HAP NP) as revealed by TEM and DLS measurements (Figure 1B(i,ii)) and Table 1. The presence of plasmin seems to make the nanocomplex smaller and this may be due to enhanced crosslinking of the complex. Further, particle charge was measured by zeta potential measurement. Both nanoparticles, with or without plasmin, exhibited slightly negative zeta potential values as expected, as the formulation contained more heparin compared to arginine. 

**Figure 2:**
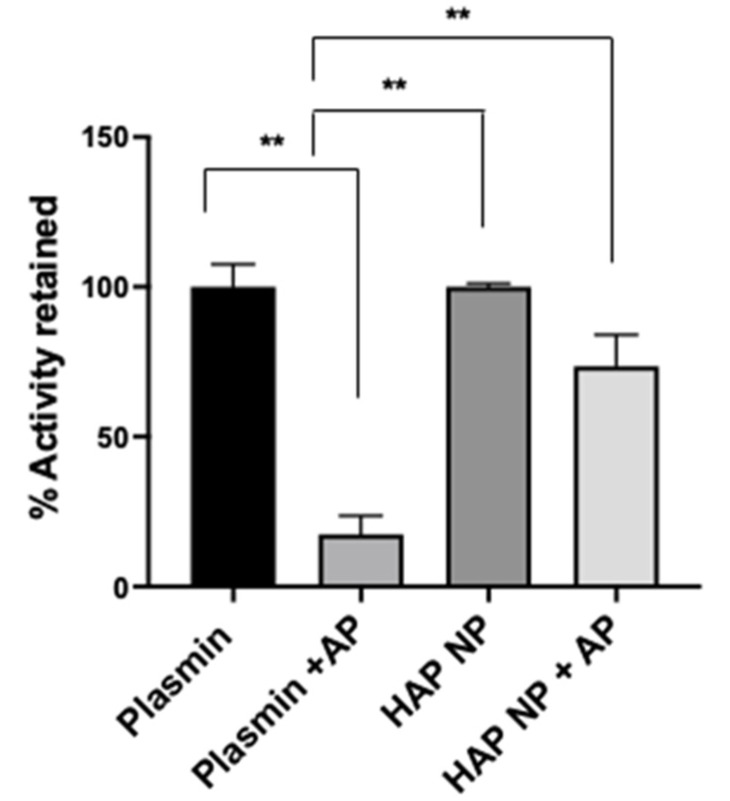
Activity of the nanoformulation and free plasmin with and without α2-antiplasmin with equimolar ratio. Plasmin activity after 30 min treatment with α2-antiplasmin (AP) at 1:1 plasmin:α2-antiplasmin molar ratio. Plasmin nanoformulations (HAP NP) retained significant proteolytic activity in the presence of AP compared to plasmin. Whereas no significant difference was observed between nanoformulation and free plasmin alone. *n* = 3 + SEM, ** *p* < 0.01.

To characterize plasmin nanoparticles, a plasmin activity assay was used to determine plasmin activity in the nanoparticles. The activity assay was performed using a fluorometric plasmin substrate (Bachem, CA) cleavage assay. After incubation with the substrate, plasmin and plasmin nanoparticle activity was measured at 370/440 nm excitation and emission. The plasmin activity in the particles was determined from the calibration curve obtained from free plasmin activity under similar conditions. Next, plasmin and nanoparticle (NP) plasmin samples were treated with and without the plasmin inhibitor α2-antiplasmin at equal molar ratio; the activity is reported in Figure 2. The results showed that the HAP nanoparticles (HAP NPs) retained significantly more activity (73 ± 10.1%) compared to that of free plasmin (17 ± 6%) in the presence of α2-antiplasmin, indicating the potential higher stability of HAP NPs in the presence of plasmin inhibitors in vivo. In addition to the activity assay, the conjugation efficiency of plasmin to the particles was found separately using FITC (Fluorescein isothiocyanate) or rhodamine labeled plasmin, and it was revealed to be around 12.5 ± 5% from the calibration curve obtained for plasmin fluorescence (data not shown). 

**Figure 3:**
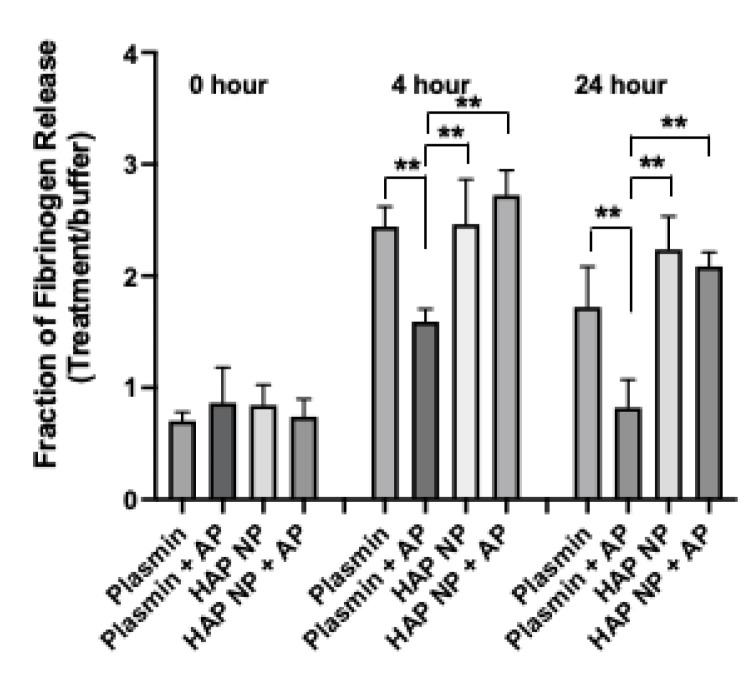
Fibrin clot lysis assay. Fibrin clot dissolution facilitated by nanoformulation and free plasmin with and without α2-antiplasmin with equimolar ratios. Fibrin clots treated with plasmin nanoformulation (HAP NP), HAP NPs and α2-antiplasmin (AP), free plasmin, free plasmin and AP, and clot lysis were measured at 0 h, 4 h and 24 h after treatment. *n* = 3 ± SEM, ** *p* < 0.01.

Next HAP nanoparticles were tested in a fibrin clot lysis assay to demonstrate their thrombolytic potential in the presence of α2-antiplasmin. Fibrin clots were made by treating fibrinogen with thrombin in 8 well or 96 well plates at 37 °C and incubated overnight. The clots were subsequently washed thrice to remove unstable gel fragments and then treated with buffer, plasmin, or HAP NPs containing equivalent plasmin concentration with or without α2-antiplasmin inhibitor in a time course experiment. The release of the FITC fibrinogen, indicative of plasmin activity, was measured immediately, and at 4 h, and 24 h after treatment. The fraction of fibrinogen release due to treatment to that of buffer was then plotted (Figure 3). HAP NPs showed significantly better clot lysis efficacy (2.73 ± 0.20) compared to free plasmin (1.59 ± 0.12) in the presence of α2-antiplasmin after 4 h or (2.1 ± 0.12) compared to free plasmin (0.8 ± 0.25) at 24 h of treatment. Not surprisingly, no significant difference in clot lysis was observed between plasmin and HAP nanoparticles alone. These results suggest that HAP NPs present superior thrombolytic potential over free plasmin in the presence of its natural inhibitor α2-antiplasmin and may allow them to show better thrombolytic activity in vivo. 

**Figure 4:**
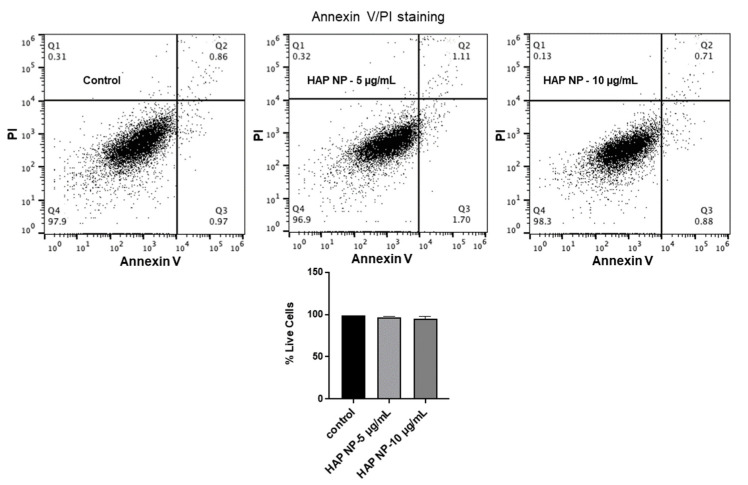
**FACS analysis of HAP NP-induced cell death.** To assess if HAP NP treatment induced toxicity and subsequent cell death, hBMVEC was treated with plasmin nanoparticles for 48 h and then stained with Annexin V FITC and PI as markers for apoptosis and necrosis and processed for FACs analysis. The four quadrants in the plots represent live cells (Q4), necrotic or late apoptotic (Q1), apoptotic cells (Q3), and dead cells (Q2). Percentage of live cells were obtained and plotted. *n* = 3 + SEM.

**Figure 5:**
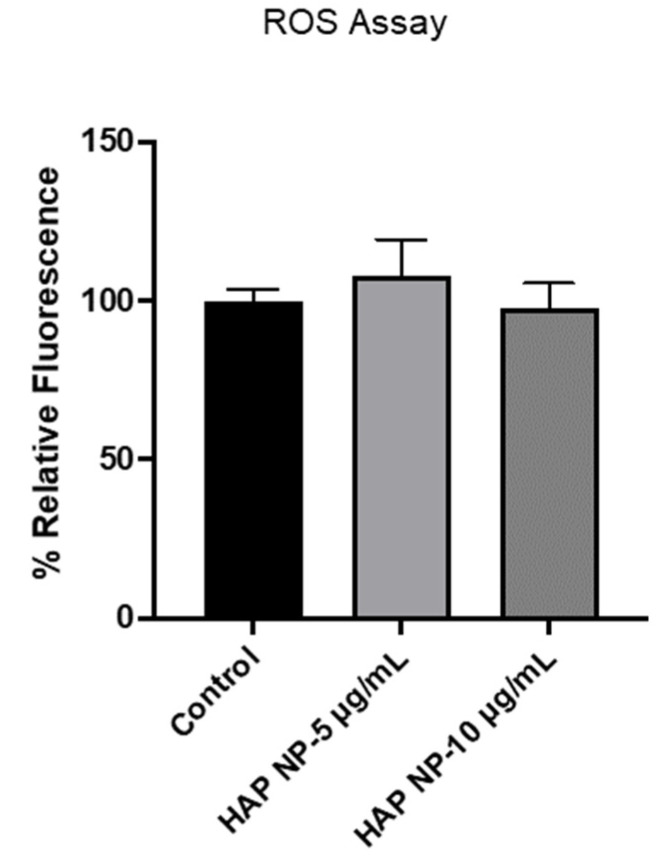
Effects of HAP nanoparticles on oxidative stress induction in hBMVEC was assessed with DCFH-DA assay. Brain endothelial cells were treated with HAP NPs (5 µg/mL or 10 µg/mL) for 48 h and cells were stained with DCFH-DA analyzed. Particles containing up to 10 µg/mL equivalent plasmin concentration showed no oxidative stress effects on the cells.

To ensure HAP nanoparticles are safe to use in vivo, these particles were then tested for their toxicity and oxidative stress effects on brain endothelial cells. We used an apoptosis/necrosis assay for the toxicity analysis since the normal metabolic toxicity assays were found not to be suitable for the study as the particles interfered with the reagent, resulting in very high metabolic readings (results not shown). As an alternative method, we used the apoptosis/necrosis assay to assess the live cells and death cell percentages due to HAP NP treatment. These assays showed that HAP NP-treated cells showed almost no cell death (>95% cell viability) compared to vehicle-treated cells and therefore could be used with minimal concerns for toxicity (Figure 4). Next, we also tested whether the particles induced any oxidative stress in hBMVECs at similar concentrations. As shown in Figure 5, no significant oxidative stress was observed in HAP NP-treated cells compared to no treatment (control) indicating a potentially safer profile for this plasmin nanoparticle formulation. 

**Figure 6:**
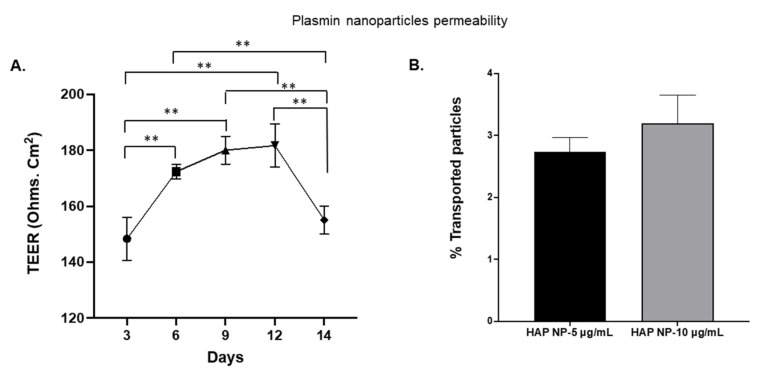
Vascular permeability of the particles was assessed in an in vitro transwell cell culture model. (**A**) TEER measurements showed the highest resistance after 9 days. (**B**) HAP NPs were incubated at 9 days and permeability of the particles was quantified compared to the initial particles concentration at the apical side. The particles showed minimal transport with less than 4% extravasation. *n* = 3 + SEM, ** *p* < 0.01.

Since plasmin extravasation and the possibility of subsequent hemorrhagic conversion is of concern in stroke therapy, we also tested whether HAP NPs would be able to cross the barrier in an in vitro transwell blood brain barrier model. Nano particles were introduced on the luminal side of the BBB 48 h after in vitro barrier establishment (Figure 6A) and permeability of the particles on the abluminal side were analyzed after 2 h of incubation. The permeability of the particles was determined based on the ratios of the nanoparticle concentrations in basolateral (abluminal, C_A_) to that of the concentrations in the apical (luminal, C_L_) side at the start of the incubation. As can be seen in Figure 6B, no significant permeability/extravasation of the particles was observed as only less than 4% of these particles could be detected on the abluminal side. The results indicate that the HAP nanoparticles might be safe to use in thrombolysis in vivo without major concerns for crossing the BBB. 

**Figure 7:**
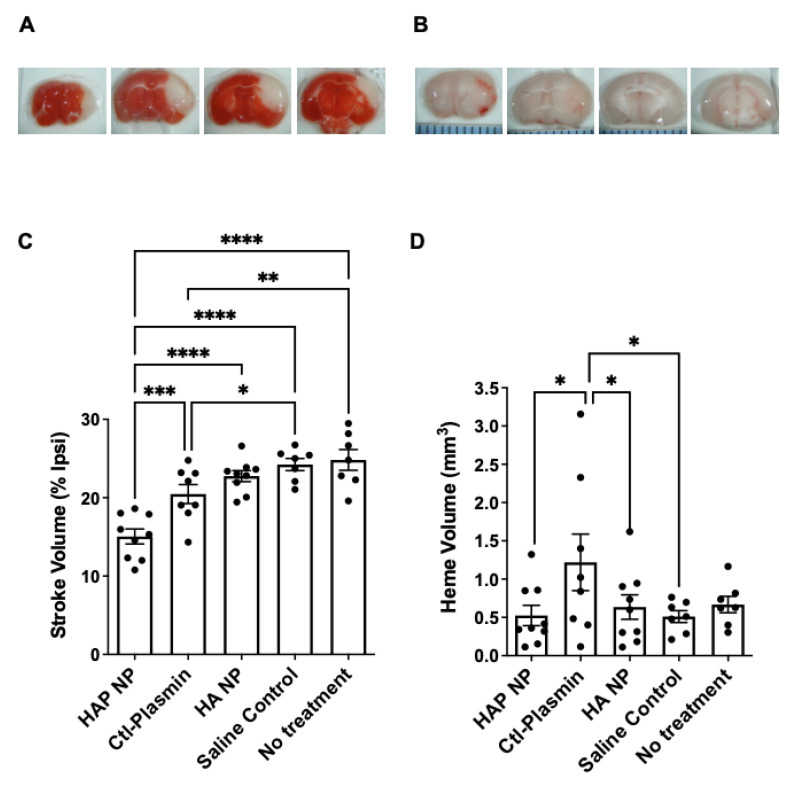
Treatment with HAP particles reduced stroke infarct volume without overt increases in hemorrhagic transformation following MCAO. (**A**) Representative images of TTC staining of coronal brain sections from one mouse 72 h after MCAO. (**B**) Representative images of coronal brain sections from one mouse showing hemorrhage 72 h after MCAO. (**C**) Wild type C57BL/6J mice were subjected to MCAO and 3 h later treated i.v. with plasmin nano particles (HAP NP, 125 µg/kg), free plasmin (Ctl-Plasmin, 125 µg/kg), nano particle control (HA NP), or saline control or No treatment. (**D**) The volume of hemorrhage was quantified from serial brain sections 72 h after MCAO. *n* = 7–9 in each group, * *p* < 0.05, ** *p* < 0.01, *** *p* < 0.001, **** *p* < 0.0001. One-way ANOVA followed by Bonferroni post-hoc test for comparison of three or more groups was used.

After characterizing the HAP nanoparticles in vitro, we next tested efficacy of these particles in an in vivo photothrombotic stroke model. The results demonstrate that treatment with HAP NPs, 3 h after middle cerebral artery occlusion (MCAO), significantly decreased stroke volume by 27% and 34% compared to free plasmin (Ctl-Plasmin) and nanoparticle controls (HA NP), respectively (Figure 7C). Interestingly, free plasmin also reduced stroke volume significantly, albeit to a much lesser extent than HAP NPs when compared to saline control (17% versus 40%, Figure 7C), suggesting much better efficacy for HAP NPs. Additionally, HAP NPs demonstrated a superior safety profile in terms of hemorrhagic conversion than that of free plasmin (0.3 mm^3^ versus 0.8 mm^3^ hemorrhage volume, respectively, Figure 7D). Finally, HAP NPs showed no signs of overt increases in hemorrhage when compared to all vehicle controls (HA NPs, saline or no treatment, Figure 7D). 

## 3. Discussion

The findings from this study show that nanotechnological applications can be used to develop more stable plasmin-based stroke therapeutics. Our study shows that heparin- arginine-based plasmin nanoparticles were able to stabilize plasmin in the presence of α2-antiplasmin compared to free plasmin. The heparin and arginine complex seems to help shield the plasmin from the surroundings thereby preventing direct exposure to plasmin protease inhibitors. Additionally, treatment using this plasmin nanoformulation showed an improved stroke outcome in a photothrombotic stroke model, indicating that it could be used as an efficient and safe thrombolytic agent compared to free plasmin treatment, without causing significant hemorrhagic risk. Hence the studies show that a nanoparticle formulation that could shield plasmin from endogenous plasmin inhibitors, and that does not cross the blood barrier, could have increased therapeutic efficacy in treating ischemic stroke. However, our current studies are still at an early stage and future studies are needed to incorporate dose–response testing, clot lysis analysis, and to assess the potential to extend the treatment window beyond the current 4.5 h limitation of thrombolysis with tPA. For instance, particle size, site-specificity, diffusivity inside the clot, fibrinolytic activity, and biocompatibility are some of the major parameters that need to be fine-tuned for optimal design. Further, novel thrombolytic strategies, such as formulations with multi-functionality or a combination therapy, may produce better outcomes. Therefore, the nanotherapeutic formulations still require more characterization and improvement to realize their full potential in treating ischemic stroke. 

Since stroke is one of the most devastating diseases, improvement in therapeutic delivery and treatment modalities would be clinically significant. Compared to tPA, plasmin nanoformulation could be a more attractive alternative due to it direct fibrinolysis and safer thrombolytic profile. The approach reported in this paper further supports using plasmin-based nanoformulations as novel therapeutics to treat ischemic stroke.

## 4. Materials and Methods

Human lys-plasmin (HPLM), human fibrinogen, and human thrombin were purchased form Molecular Innovations., Novi, MI. Plasmin substrate was purchased from Bachem, Torrance, CA. The apoptosis and necrosis assay kit was purchased from Biotium, Fremont, CA. Human α2-antiplasmin, and all the other chemicals, were purchased from Millipore Sigma, St. Louis, MO, or Thermo Fisher Scientific. 

### 4.1. Plasmin Nanoparticle Formulation

Heparin (cat no. H3393, Sigma Aldrich, St. Louis, MO, USA) and polyarginine (PLA) (P4663, Sigma Aldrich) was used for the plasmin nanoformulation. First stock solutions of 5 mg/mL of heparin, 1 mg/mL of PLA, 100 mM EDC, 100mM NHS were prepared at pH3 in 100 mM HEPES with 100 mM NaCl buffer. Then 5 mg/mL of heparin was first reacted with 1m M of EDC, and NHS for 20 min. Next human lys-plasmin (1 mg/mL) in HEPES pH 7.4 with twice the volume of the heparin was added to the solution and the reaction was continued for another 1.5 h at 4 °C. After the reaction, 1 mg/mL of polyarginine with an equal volume to that of heparin was added and the total mixture was allowed to react overnight. The following day, heparin-arginine-plasmin (HAP) nanoparticles were centrifuged and purified, and a physicochemical characterization was performed. The amount of plasmin in the nanoparticles was confirmed by FITC or rhodamine fluorescence. Briefly, the nanoparticles were formed with plasmin-FITC or plasmin-rhodamine, and the conjugation efficiency was determined by a calibration standard curve of the FITC fluorescence (491/519 nm excitation/emission) or rhodamine fluorescence (552/575 excitation emission) using a plate reader in the laboratory. The activity of the plasmin was determined using plasmin activity assay and the physicochemical characterizations were performed to determine the size, zetapotential, and morphology. 

### 4.2. TEM, DLS and Zeta Potential 

Nanoparticle size and morphology were characterized by transmission electron microscope (TEM), and dynamic light scattering (DLS). For TEM characterization, 5 µL of the particles was placed on formvar/carbon 200 mesh copper grids (EMS, Hatfield, PA, USA), negatively stained with 2% phosphotungstic acid, pH 7.4, and imaged at the UM Ann Arbor core facility. DLS and zeta potential measurements were performed using a Malvern particle sizer in the lab. Particles were diluted 10x times prior to measurements. Experiments were repeated for four different samples to achieve sufficient statistical data. 

### 4.3. Plasmin Activity

Plasmin activity was measured by fluorogenic substrate cleavage. Plasmin substrate (Bachem, Fremont, CA, USA) at a concentration of 0.1 mM was used and the cleavage of the substrate due to free plasmin, and plasmin nanoparticles was determined at 370/440 nm excitation and emission using a spectramax M3 plate reader. Nanoformulation activity was compared to that of equivalent plasmin activity with and without anti-plasmin at equimolar concentration. 

### 4.4. Fibrin Clot Lysis Assay

Fibrin clots were made from 5 mg/mL FITC-fibrinogen, 10 units/mL of thrombin, and 20 mM CaCl_2_ at 37 °C overnight. Next, clots were washed with buffer and subsequently treated with plasmin nanoformulation and free plasmin with and without α2-antiplasmin consisting of 10 µg/mL plasmin. Clot dissolution was monitored by the FITC-fibrinogen fluorescence released at 491/519 nm excitation emission. The release was recorded immediately, and at 4 h and 24 h time points from the treatment for three independent experiments. 

### 4.5. Permeability Assay

To assess whether the plasmin nanoparticles would extravasate through the blood brain barrier, an in vitro transwell model of the blood brain barrier was used. Human brain microvascular endothelial cells (hBMVECs) were cultured in medium containing M-199, supplemented with 10% FBS, and 5% Pen-Strep on the upper chamber for several days to allow tight junction formation. Endothelial cells at a density of 100,000 cells/well was seeded in the upper chamber of a 24 well plate insert with a membrane pore size of 3 μm. TEER values were recorded using an EVOM2 volt-ohmmeter (World Precision Instruments, Sarasota, FL, USA). The measurements revealed maximum TEER values around 9 days after culture. On the 9th day, particles were added, and after 2 h, transport of the particles to the lower chamber was measured by the fluorescence of the FITC-plasmin nanoparticles. The percentage of particles extravasated into the lower chamber to that of the particles in the upper chamber at the beginning of the permeability process was quantified. Experiments were repeated three times to obtain statistical significance. 

### 4.6. Apoptosis-Necrosis Assay

To test the toxicity effects of the particles to brain endothelial cells, human brain microvascular endothelial cells were cultured in 24 wells at a density of 5 × 10^4^ cells/well for 24 h. Cells were then treated with, free plasmin, plasmin nanoparticles containing 5 µg/mL, and 10 µg/mL of plasmin. The plasmin concentrations were chosen based on previous studies with plasmin and tPA in cell culture studies [30,31]. The cells were subsequently incubated for another 48 h. Cells were then stained for apoptosis and necrosis according to the manufacturers protocol. The percentages of live cells, apoptosis, and necrosis of the cells with and without the treatment were then quantified by flowcytometry using an Attune NXT flow cytometer in the lab. 

### 4.7. ROS

To assess whether the plasmin nanoparticles induce oxidative stress, H2DCFDA assay (Thermo Fisher Scientific, Waltham, MA, USA) was used. Several studies have reported oxidative stress quantification by 2,7-dichlorodihydrofluorescein (DCFH)-based fluorescent probes [20,21]. For the study, hBMVEC cells were plated in 96 wells at a density of 10^4^ cells/well and were cultured overnight in medium containing M-199, supplemented with 10% FBS, and 5% Pen-Strep. Cells were then treated with plasmin nanoparticle or free plasmin containing (5 µg/mL, and 10 µg/mL of plasmin) for 48 h. DCFH-DA assay fluorescence was performed at 495/527 nm excitation and emission. Four independent experiments were performed, and the average readings were obtained.

### 4.8. Statistical Analysis

Data was collected from three or more replicates for each experiment. The data are presented as mean and standard error of the mean (SEM). *p*-values were determined from the results of at least 3 independent experiments. Statistical significance was determined by analysis of variance (ANOVA) followed by Tukey’s HSD post-hoc analysis or unpaired student t-tests. Results with significance are presented as ns: non-significant, * *p* < 0.05, ** *p* < 0.01, *** *p* < 0.001, **** *p* < 0.0001.

### 4.9. Animals

All animal procedures were approved by and carried out in accordance with the guidelines of the Institutional Animal Care and Use Committee at the University of Michigan. Mice were housed under a 12-h light/dark cycle with free access to water and a standard rodent chow. Male C57BL/6J mice (10–12 weeks old) were obtained from Jackson Laboratories. Animals were anesthetized using chloral hydrate (450 mg/kg, I.P) unless noted otherwise.

### 4.10. Middle Cerebral Artery Occlusion (MCAO)

Photothrombotic MCAO was induced as described (Su et al. 2008, 2017). Briefly, male wild-type, c57BL/6J mice (Jackson Lab, 10–12 weeks old) were anesthetized with chloral hydrate (450 mg/kg Fisher Scientific) and placed securely under a dissecting microscope. The left MCA was exposed, and a laser Doppler flow probe (Type N, Transonic Systems) was placed on the surface of the cerebral cortex 1.5 mm dorsal median from the bifurcation of MCA. The probe was connected to a flowmeter (Transonic model BLF21) and tissue perfusion units (TPU) recorded with continuous data acquisition (Windaq, DATAQ). A 3.5-mW green laser (540 nm, Melles Griot) was directed at the MCA from 6 cm, and Rose Bengal (RB) (Fisher) 10 mg/mL in Lactate Ringer’s solution was injected via the tail vein (50 mg/kg). The TPU was recorded, and stable occlusion was achieved when the TPU dropped to less than 20% of pre-occlusion levels for 20 min and did not rebound within 10 min after laser withdrawal. Based on power calculations, each group had 7-9 animals. Additionally, we observed zero mortality and no animal was excluded from the study. 

### 4.11. Stroke Volume Measurement

For assessment of infarct volume, mice were sacrificed at 72 h after stroke and brains were removed and cut into 2 mm thick coronal sections and stained with 4% 2,3,5-triphenyltetrazolium chloride (TTC) in PBS for 20 min at 37 °C, and then fixed in 4% paraformaldehyde solution for 10 min. Five brain slices/mouse were analyzed using the Image J software (NIH) by an investigator blind to the treatments. The following formula was used to calculate stroke volume:

V%stroke = ∑(Areas of lesion)/∑(Areas of ipsilateral hemisphere) * 100

Stroke volume (V%stroke) was calculated as percent of the ipsilateral hemisphere in order to avoid an artifact due to brain edema.

### 4.12. Heme Volume Measurement

For assessment of hemorrhagic volume in the brain, digital images (both sides) were captured (using the same coronal sections in TTC staining) before brain sections were fully developed in TTC solutions. Images were analyzed using the Image J software (NIH) by an investigator blind to the treatments as described in (Su et al. 2017). The following formula was used to calculate hemorrhagic volume:

V_ICH_ = (∑ (Areas of ICH(mm^2^)/2) × 2 mm

where V_ICH_ is ICH volume calculated in cubic millimeters.

## Figures and Tables

**Table 1:** Nanoparticle size and zeta potential.

Nanoparticle	Zavg (nm)	Zeta Potential (mv)
HP NP	487.46 ± 19.71	−20.17 ± 7.60
HAP NP	377.85 ± 17.81	−33.86 ± 0.82

## Data Availability

Not applicable.
